# Association analyses of repeated measures on triglyceride and high-density lipoprotein levels: insights from GAW20

**DOI:** 10.1186/s12863-018-0651-6

**Published:** 2018-09-17

**Authors:** Saurabh Ghosh, David W. Fardo

**Affiliations:** 1 Human Genetics Unit, Indian Statistical Institute, 203 B.T. Road, Kolkata, 700108 India; 2 Department of Biostatistics, University of Kentucky, 111 Washington Ave, Lexington, KY 40536 USA

**Keywords:** Genome-wide association, Epigenome-wide association, Longitudinal data, Linear mixed models, Quasi-likelihood, Multivariate phenotypes

## Abstract

**Background:**

The GAW20 group formed on the theme of methods for association analyses of repeated measures comprised 4sets of investigators. The provided “real” data set included genotypes obtained from a human whole-genome association study based on longitudinal measurements of triglycerides (TGs) and high-density lipoprotein in addition to methylation levels before and after administration of fenofibrate. The simulated data set contained 200 replications of methylation levels and posttreatment TGs, mimicking the real data set.

**Results:**

The different investigators in the group focused on the statistical challenges unique to family-based association analyses of phenotypes measured longitudinally and applied a wide spectrum of statistical methods such as linear mixed models, generalized estimating equations, and quasi-likelihood–based regression models. This article discusses the varying strategies explored by the group’s investigators with the common goal of improving the power to detect association with repeated measures of a phenotype.

**Conclusions:**

Although it is difficult to identify a common message emanating from the different contributions because of the diversity in the issues addressed, the unifying theme of the contributions lie in the search for novel analytic strategies to circumvent the limitations of existing methodologies to detect genetic association.

## Background

Even though genome-wide association studies (GWAS) have successfully identified novel genetic variants that confer risk to various complex disorders, the proportion of trait variance that can be explained by the identified variants remains abysmally low compared to the estimates of heritability of these traits obtained from twin studies. It has been argued that analyzing quantitative precursors of a clinical end-point trait, which carry more information on interindividual phenotypic variability compared to an often binary end-point trait, may be a more powerful strategy to unravel the genetic architecture of the underlying complex disorder [1]. However, quantitative trait values vary over time; consequently, measurements at a single time point may not serve as optimal phenotypes in genetic association analyses. Longitudinal data are known to contain more information on the genetic and environmental factors modulating a phenotype compared to cross-sectional studies [2]. Moreover, there is increasing belief that epigenetic factors, such as methylation and histone acetylation, may be able to explain some of the “missing heritability” in these complex traits.

The major statistical challenge encountered in the genetic association analyses of repeated phenotype measurements is the modeling of the phenotype values across the different time points. Cross-sectional analyses of phenotype values at different time points may not only provide inconsistent inferences but also can exacerbate the problem of multiple testing inherent in GWAS. On the other hand, a naïve multivariate analysis ignoring the correlation structure of phenotype values across the different time points may result in power loss [3]. It is also challenging to develop methodologies that can incorporate multiple phenotypes in the longitudinal framework. Likelihood-based methods, such as variance components [4, 5] for analyzing multivariate phenotypes, may be directly adopted to analyze a single phenotype in a longitudinal framework, but such methods are, in general, sensitive to violations of the underlying assumptions used for modeling the vector comprising the phenotype values at the different time points. Alternatively, data reduction techniques, such as principal components analysis [6], circumvent the problem of robustness, but often yield reduced power to detect associations. The family-based framework involves an additional challenge of modeling phenotypic, familial, and serial correlations, but this can be addressed, for example, by latent variable methodology that incorporates both longitudinal and family correlation [7].

The GAW20 provided an excellent opportunity to explore the various statistical issues pertaining to association analyses of longitudinal phenotypes, whole-genome single-nucleotide polymorphism (SNP) data, and methylation (cytosine-phosphate-guanine [CpG]) data. The real data provided in GAW20 includes pedigrees from the Genetics of Lipid Lowering Drugs and Diet Network (GOLDN) study comprising 1105 participants and including genome-wide information on 597,145 variant sites and methylation levels at 463,995 CpG sites. Repeated measurements are available on 2 phenotypes: triglyceride (TG) levels and high-density lipoprotein (HDL) levels, measured twice each before and after the intervention of fenofibrate, a blood lipid-lowering drug. Information was also available on a few additional variables, such as age, sex, smoking status, and study center. The simulated data provided in GAW20 was created to mimic the real data and assumed 5 “major gene” causal SNP effects. Each of these effects on TG response was modeled to be proportional to the degree that the physically closest CpG site is unmethylated. Two hundred simulation replicates of posttreatment TG and methylation levels were generated under this growth-curve-based pharmacoepigenetic response model [8].

There were 4 contributions from our group, of which 3 considered the real data while 1 [9] analyzed the simulated data. Table 1 summarizes the data analyzed in the different contributions. The contributions that focused only on detecting association between SNPs and longitudinal phenotypes considered a minor allele frequency threshold of greater than 0.05 based on the common variant–common disease hypothesis. While Das et al. [10] and Kulkarni et al. [11] performed genome-wide association analyses based on TG and HDL levels, Wei and Wu [12] carried out an epigenome-wide association analysis based on TG and methylation levels, and Strickland et al. [9] restricted their analyses to 10 SNPs along with the nearest CpG sites in the simulated data set (5 causal SNPs that were simulated to exhibit large effects on TG levels and 5 noncausal SNPs). The association analyses in the different contributions adopted variance components approaches, linear mixed effects models, generalized estimating equations, quadratic inference functions, or tests based on transmission disequilibrium. None of the contributions performed analyses of cross-sectional phenotype values exclusively. Although there were varying statistical issues addressed by the different investigators in the group, we were able to integrate these issues into a few unifying themes as discussed in the subsequent sections.

**Table 1:** Description of the data analyzed in the different contributions

Contribution	Type of data analyzed	Phenotypes analyzed
Das et al	GOLDN (GWAS)	TG, HDL
Kulkarni et al	GOLDN (GWAS)	TG (adjusted for HDL)
Wei and Wu	GOLDN (EWAS)	TG, methylation
Strickland et al	Simulated (10 SNPs)	TG, methylation

EWAS epigenome-wide association study, GOLDN Genetics of Lipid Lowering Drugs and Diet Network, HDL high-density lipoprotein, SNP single-nucleotide polymorphism, TG triglyceride

## Methods

### Combining repeated measurements of phenotypes

A primary challenge in analyzing a phenotype in a longitudinal framework lies in the integration of the phenotype values across different time points in a multivariate phenotype vector or a reduced univariate phenotype. All the contributions used logarithmic transformations on phenotypic values to induce normality. Das et al. [10] considered an 8-dimensional phenotype vector comprising the 4 measurements of TG and the 4 measurements of HDL while performing the GWAS with the 2 quantitative traits. In a separate analysis, they considered the difference in the means of the 2 measurements of TG levels made before administration of fenofibrate with those made after treatment as the phenotype of interest to analyze the effect of fenofibrate on genomic associations. Strickland et al. [9] and Wei and Wu [12] also summarized the TG measurements pre- and postadministration of fenofibrate by the respective means. Kulkarni et al. considered the first principal component of the TG measurements before and after the administration of fenofibrate as a summarized phenotype in their model. Because the different contributions considered diverse alternatives of combining the phenotype values, comparisons of the performances based on the different choices were not performed and hence, an optimal method of integrating the phenotype values was not investigated.

### Adjustment for covariates

Environmental confounders as well as clinically correlated endophenotypes are known to adversely affect inferences on genetic association [13], especially with respect to false-positive rates. Thus, it is necessary to adjust the effects of these factors from the phenotypes of interest to assess the true effects of the genetic variants. Data were available on 5 covariates: HDL levels, age, sex, smoking status, and study center. Even though Das et al. [10] considered HDL levels jointly with TG levels as the primary phenotype, they included age and smoking status as fixed effects in the regression model. Strickland et al. [9], as well as Wei and Wu [12], used a similar method of adjustment by modeling age, sex, study centers, and methylation levels at CpG sites as fixed effects. Wei and Wu [12] also included treatment (ie, prestatus or poststatus of fenofibrate administration) as a binary covariate in the model. Kulkarni et al. [11] used a generalized linear regression of TG levels on HDL levels to obtain TG levels adjusted for HDL levels.

Das et al. [10] and Kulkarni et al. [11] used different strategies to impute missing phenotypic values to maximize the information on longitudinal data. Kulkarni et al. [11] used an unrelated set of founders from the pedigrees to estimate the missing phenotype values based on data on the available phenotype values using an expectation maximization algorithm under the assumption of multivariate normality of phenotype values and then used the plug-in estimates of the parameters to impute the missing phenotype values of the nonfounders in the pedigrees. Das et al. [10] also used the expectation maximization algorithm but under their assumed mixed model framework. The imputed values corresponding to the missing phenotypic observations are computed under the null hypothesis of absence of any SNP effect and zero genetic correlation.

### Statistical methods for association

The different contributions explored contrasting statistical approaches to analyze TG levels before and after the administration of fenofibrate. The most popular approach to model the longitudinal data in the family-based framework is to use linear mixed effects models, primarily because such models can both account for relatedness within families and correct for population stratification between families via incorporation of principal components [14]. Moreover, the flexibility of modeling the effect of fenofibrate both as a main effect and as an interaction effect with SNPs results in higher powers of detecting association. However, analyses based on linear mixed models are susceptible to violations in distributional assumptions (such as normality). On the other hand, transmission-based tests for association that model transmission probabilities of parental alleles conditioned on offspring phenotypes require partitioning large pedigrees into nuclear families, resulting in possible loss of power but are relatively more robust with respect to model misspecifications as the tests are conditioned on phenotype values and hence do not require modeling the correlation structure of the repeated measurements of a phenotype across different time points. Moreover, such models can incorporate both quantitative and qualitative components in the vector of measurements [15, 16].

Strickland et al. [9] considered 2 outcome variables: TG change scores calculated as the difference between the posttreatment and pretreatment average log-transformed TG levels and TG adjusted by baseline TG to compare the performances of 3complementary multivariate methods that account for the correlation induced by familial relatedness: linear mixed effects models, generalized estimating equations, and quadratic inference functions. Wei and Wu [12] et al. employed a mixed effects model with a familial random effect and conducted an epigenome-wide association study. They used a time-by-methylation interaction test to identify candidate genes for a gene-set enrichment analysis. Das et al. [10] considered a multivariate phenotype vector comprising TG and HDL levels, each measured at 4 different time points and used a linear mixed model to perform a GWAS with the set of SNPs. They also used a modification of Henderson’s mixed model to develop a multilocus association test with the mean change in TG levels caused by the administration of fenofibrate. Kulkarni et al. [11] developed a novel test for association based on quasi-likelihood where the conditional distribution of the transmission indicators of alleles from both parents (of whom at least 1 must be heterozygous) at a SNP is modeled using a logistic link function of the principal components of TG levels (both unadjusted and adjusted for HDL levels) before and after the administration of fenofibrate.

## Results

Because contrasting statistical methodologies were applied to analyze different subsets of the GAW20 data on repeated measurements of TG and HDL levels, it was not feasible to explore the extent of similarity in the results obtained by the different investigators in the group. The phenotype definitions as well as the objectives with respect to these definitions were also not strictly comparable. Thus, it is not unexpected that the significantly associated SNPs obtained by Das et al. [10] and Kulkarni et al. [11] are located in different genomic regions. With respect to objectives of individual contributions in the group, we summarize the major conclusions as follows: Das et al. [10] showed that testing for association at the gene level (ie, simultaneously testing at multiple SNPs) may significantly reduce the multiple testing burden compared to single SNP association tests. Kulkarni et al. [11] observed that including transmission information from both parents may increase the likelihood of obtaining a larger number of significant association findings compared to classical transmission-based tests that consider transmissions only from heterozygous parents. Wei and Wu [12] demonstrated that integrating information on association obtained from methylation data and from pathway databases is likely to yield a larger set of putative genes modulating the phenotype of interest. Based on the empirical error rates obtained from the 200 simulated replications, Strickland et al. [9] found that while tests based on linear mixed effects models maintained the appropriate size, those based on the generalized estimating equations or the quadratic influence function have inflated false positive rates unless an explicit bias correction is employed. A common observation in all the contributions was the lack of power in obtaining a larger number of true association findings as a consequence of inadequate sample sizes.

## Discussion

A majority of the contributions [9, 10, 12] highlighted the flexibility of linear mixed effects models, which can be used to model the dependence of TG values across different time points in a family-based framework. On the other hand, the transmission-based test using a quasi-likelihood model [11] provides a semiparametric alternative that is likely to be more robust to violations in distributional assumptions.

Although it is difficult to determine whether novel association findings obtained from real data sets are indeed true positives, the probability of the finding to be a false positive is reduced if contrasting statistical methods provide significant evidence of association in similar genomic regions in the absence of confounding effects. Because the phenotype definitions were not identical in the different contributions, it was difficult to evaluate the consistency of the different analytical strategies in yielding similar association findings. However, all contributions that analyzed the GOLDN data [10–12] obtained significant association findings in genes or CpG sites that were previously reported to be involved in heart-related disorders, lending additional credibility to the approaches taken.

Finally, we wish to highlight that a major issue corresponding to any novel methodology is the computational burden involved in the analysis. Even though the transmission-based test using the quasi-likelihood approach requires partitioning of large pedigrees into nuclear families, it is not computationally expensive. On the other hand, large pedigrees can be directly incorporated in linear mixed effects models that assume the genetic and phenotypic correlation structures between individuals within a pedigree, but, being likelihood-based in nature, such methods are often susceptible to inflated false positives when underlying distributional assumptions on cross-sectional as well as time-dependent correlation structures on phenotype values are violated. It may be more appropriate to use empirical thresholds (one can randomly assign genotypes to parents based on estimated allele frequencies and use Mendelian transmission rules to determine offspring genotypes keeping the phenotype values unaltered so as to preserve the observed phenotypic correlation within each family) rather than asymptotic thresholds to determine the significance of a test statistic value to increase the robustness of the test. However, such a strategy may prohibitively increase the computational complexity of the methods.

## Conclusions

The common aim of the group was to explore powerful statistical methodologies for identifying genetic variants modulating triglyceride levels in a longitudinal framework. Despite the varied statistical approaches to model repeated phenotype measurements, the crucial paradigm unifying the contributions was that analyzing repeated phenotype measurements may be a more powerful strategy compared to cross-sectional analyses for identifying genetic and epigenetic factors underlying a complex trait.

## Abbreviations

CpG: cytosine-phosphate-guanine
GAW: Genetic Analysis Workshop
GOLDN: Genetics of Lipid Lowering Drugs and Diet Network
GWAS: genome-wide association studies
HDL: high-density lipoprotein
SNP: single-nucleotide polymorphism
TG: triglycerides
